# Lipid Nanoparticles for the Posterior Eye Segment

**DOI:** 10.3390/pharmaceutics14010090

**Published:** 2021-12-31

**Authors:** Lorena Bonilla, Marta Espina, Patricia Severino, Amanda Cano, Miren Ettcheto, Antoni Camins, Maria Luisa García, Eliana B. Souto, Elena Sánchez-López

**Affiliations:** 1 Department of Pharmacy, Pharmaceutical Technology and Physical Chemistry, Faculty of Pharmacy, University of Barcelona, 08028 Barcelona, Spain; lbonilla95@ub.edu (L.B.); m.espina@ub.edu (M.E.); acanofernandez@ub.edu (A.C.); marisagarcia@ub.edu (M.L.G.); 2 Institute of Nanoscience and Nanotechnology (IN2UB), University of Barcelona, 08028 Barcelona, Spain; 3 Industrial Biotechnology Program, University of Tiradentes (UNIT), Av. Murilo Dantas 300, Aracaju 49032-490, Brazil; patricia_severino@itp.org.br; 4 Centro de Investigación Biomédica en Red de Enfermedades Neurodegenerativas (CIBERNED), University of Barcelona, 08028 Barcelona, Spain; mirenettcheto@ub.edu (M.E.); camins@ub.edu (A.C.); 5 Department of Pharmacology, Toxicology and Therapeutic Chemistry, Faculty of Pharmacy and Food Sciences, University of Barcelona, 08028 Barcelona, Spain; 6 CEB—Centre of Biological Engineering, Campus de Gualtar, University of Minho, 4710-057 Braga, Portugal

**Keywords:** ocular drug delivery, ocular barriers, lipid nanoparticles, posterior segment, drug transport, NLC, SLN

## Abstract

This review highlights the application of lipid nanoparticles (Solid Lipid Nanoparticles, Nanostructured Lipid Carriers, or Lipid Drug Conjugates) as effective drug carriers for pathologies affecting the posterior ocular segment. Eye anatomy and the most relevant diseases affecting the posterior segment will be summarized. Moreover, preparation methods and different types and subtypes of lipid nanoparticles will also be reviewed. Lipid nanoparticles used as carriers to deliver drugs to the posterior eye segment as well as their administration routes, pharmaceutical forms and ocular distribution will be discussed emphasizing the different targeting strategies most recently employed for ocular drug delivery.

## 1. Introduction

Ocular diseases associated with the posterior segment of the eye may lead to visual impairment, which ultimately might result in blindness. Today, more than 100 million people suffer from ocular pathologies or even blindness due to a posterior ocular disease [1,2]. The effectiveness of commercial drugs and active compounds under development is limited by the delivery of these drugs into the inner tissues. Only less than 5% of the administered traditional ocular eye drops are effectively absorbed into the eye, reaching the inner ocular tissues. This fact is due to restrictive ocular barriers that protect the eye against penetration of external substances through different barriers such as the cornea and the blood retinal barrier, and also due to effective elimination mechanisms such as tear turnover and drainage [3].

In the recent years, ocular drug delivery systems were investigated to improve ocular retention and drug absorption. Furthermore, they can act as controlled release systems that reduce administration frequency. These drug delivery systems include hydrogels, nanomicelles, dendrimers, polymeric nanoparticles, nanosuspensions, liposomes and lipid nanoparticles [4,5]. Drug delivery systems based on nanocarriers are highly interesting due to their small particle size, low ocular irritation potential and suitable drug availability. Moreover, for topical drug delivery, the nanocarrier should overcome the mucous layer of the corneal surface. Therefore, nanocarriers made of polymers or lipids with mucoadhesive properties are of particular relevance [6]. Among other nanocarriers, lipid nanoparticles constitute one of the most relevant systems due to their unique characteristics and their multiple advantages compared to other drug delivery approaches such as liposomes or polymeric nanoparticles [6,7].

## 2. Ocular Anatomy

The human eyeball consists of a spherical three-layer structure comprised of the sclera which constitutes the outer fibrous layer, the uvea, corresponding to the middle vascular layer, and the retina being the inner nervous tissue layer [3]. Moreover, the eye is divided into an anterior and posterior segment. The anterior segment contains the cornea, conjunctiva, iris, ciliary body, the lens, the lachrymal apparatus, eyelids and the anterior and posterior chambers. The posterior segment is behind the lens and contains the sclera, choroid, retina, vitreous humor and optic nerve [8].

### 2.1. The Anterior Segment: Barriers Implicated to Arrive to the Posterior Segment

**Figure 1:**
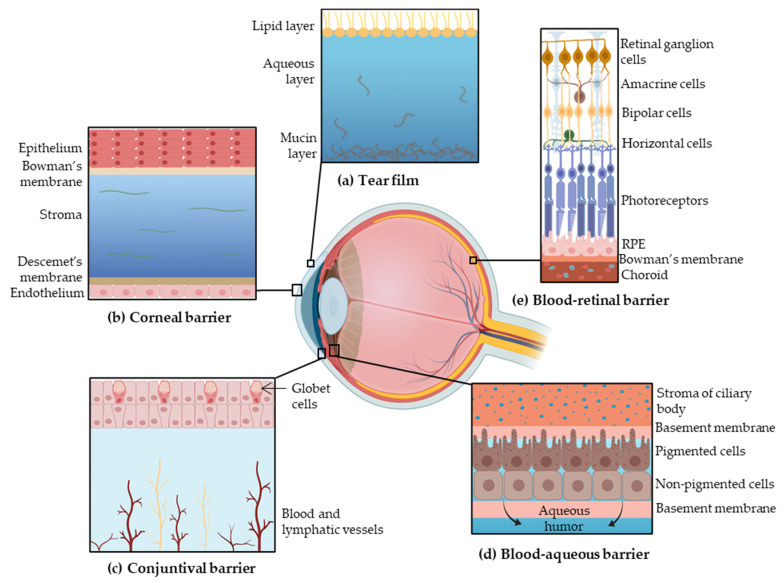
Barriers to ocular drug penetration: (**a**) the tear film is composed of three layers, the lipid layer, the aqueous layer and the mucin layer; (**b**) the corneal layer is formed by epithelium, Bowman’s membrane, stroma, Descemet’s membrane and endothelium; (**c**) the conjunctival barrier is vascularized; (**d**) the blood–aqueous barrier starts on the stroma of ciliary body and is composed by the basement membrane, pigmented cells, nonpigmented cells and delimited by the basement membrane; (**e**) the blood–retinal barrier is formed by the retinal ganglion cells, amacrine cells, bipolar cells, horizontal cells, both types of photoreceptors, retinal pigment epithelium (RPE) and the Bowman’s membrane. Adapted from [3,14,15], Sánchez-López, E., 2017; Farid, R.M., 2017; and Keeling, E., 2018.

Topical administration is the most convenient, self-administrable and non-invasive route for ocular drug delivery in superficial diseases, such as infection or inflammation. Drug transport into internal tissues of the eye via corneal/noncorneal routes involves some complex processes and different barriers, which reduce the bioavailability of the administrated drugs [9]. These eye barriers have two main functions: regulate ocular microenvironment and maintain the transparency of the anterior segment, in addition to separate this portion from blood entering the eye. The main barriers for ocular drug delivery are the tear film, the corneal barrier, the conjunctival barrier, post-corneal area and efflux transporters (Figure 1) [10].

#### 2.1.1. Tear Film

The tear film is the first obstacle for topically administered drugs due to the high turnover. The tear film is composed by three layers: an external lipid layer, an aqueous middle layer and the internal mucin layer. This film is mainly composed of water, electrolytes and proteins [11]. Under normal conditions, 16% of the tear film is renewed every minute, contributing to drugs removal [4].

Furthermore, the majority of the administrated volume is drained from the conjunctival sac into the nasolacrimal duct or cleared from precorneal area [9]. About 95% of the dose administrated is eliminated systemically by the conjunctiva and nasolacrimal duct. The walls of the lacrimal sac and nasolacrimal duct are vascularized, promoting the systematic absorption of the drug, which may cause secondary effects [12].

#### 2.1.2. Corneal Barrier

The cornea constitutes a physical barrier limiting the penetration of drugs. It is a highly organized tissue composed of five layers: epithelium, Bowman’s membrane, stroma, Descemet’s membrane and endothelium. The superficial corneal epithelium is composed of multiple layers of stratified squamous non-keratinized epithelial cells. The surface of the epithelium is covered by a complex glycocalyx composed of glycoproteins, mostly mucins, which confers a negative charge at physiological pH [3,4]. The corneal epithelium prevents the diffusion of hydrophilic drugs through the cornea due to the presence of intercellular tight junctions (zonula occludens) and its lipophilic character [11]. In contrast, lipophilic drugs can diffuse through the epithelium by intracellular route [4]. Furthermore, the corneal stroma constitutes a highly hydrophilic tissue due to the fact that it is comprised of collagenous fibrils and glycosaminoglycans, which restricts the penetration of lipophilic drugs [11,13]. The corneal endothelium is a monolayer of cells, which is responsible for maintaining physiological corneal hydration and allows the diffusion of macromolecules into the aqueous humor. The Bowman’s membrane and Descemet’s membrane may not be as relevant in drug penetration [9,13].

#### 2.1.3. Conjunctival Barrier

Drugs can be absorbed into the anterior segment through the conjunctival/scleral route. The conjunctiva is a vascularized tissue that lines the inside of the eyelids and covers the sclera [13]. The external epithelial cells of conjunctiva form junctions limiting paracellular drug penetration. Conjunctiva is a rate-limiting barrier for permeation of hydrophilic drugs due to rapid drug elimination by conjunctival blood and lymphatic flow. Therefore, only hydrophobic drugs with low molecular weight are able to penetrate across the conjunctiva [3,11]. After overcoming conjunctival clearance, drugs can arrive to the sclera, which possesses higher permeability than the cornea. This scleral permeation rate depends on the molecular weight of the drug rather than its lipophilicity. Generally, molecules with ≥70 kDa can penetrate the sclera [3,13].

#### 2.1.4. Post-Corneal Area

Once the drug reaches the anterior eye chamber, it can be binded to melanin pigments in the uvea (iris and ciliary body), reducing its bioavailability [9]. This binding is quite unspecific, but alkaline and lipophilic drugs are expected to bind to melanin. This association is originated by Van der Waal forces, electrostatic interaction or simple charge transfer [3,16].

Furthermore, in the post-corneal area, the blood–aqueous barrier (BAB) protects the anterior and posterior segments of the eye, controlling the transport of solutes between both chambers. The BAB is placed on the ciliary body epithelium and the tight capillary endothelium of the iris. The ciliary body is a bilayer comprised of two types of cells: a pigmented cell layer (controls the movement of macromolecules from the lumen of the iris vessels into the iridial stroma) and a nonpigmented cell layer (protects the posterior chamber from circulating macromolecules) [3,17].

#### 2.1.5. Efflux Transporters

Drug delivery to the posterior segment can be achieved by passive diffusion, facilitated diffusion or by active transport. The transporters are located on the apical or basolateral cell membranes, and they are able to restrict drug absorption. The cornea and conjunctiva have efflux protein transporters such as P-Glycoprotein (P-gp), breast cancer-related protein, and multidrug resistant protein (MDR cassette) on the apical surface, which are involved in drug efflux from the cell cytoplasm leading to low bioavailability [3,12,13].

### 2.2. The Posterior Segment

Only low drug levels of the topically administered dose achieve the retina and the vitreous humor; for this reason, other administration routes, such as intravitreal, were developed. Furthermore, when the drug reaches the posterior segment, it has to overcome the blood–retinal barrier (BRB), which protects the retina from external substances (Figure 1) [9,18].

#### 2.2.1. Anatomy of the Posterior Segment

**Figure 2:**
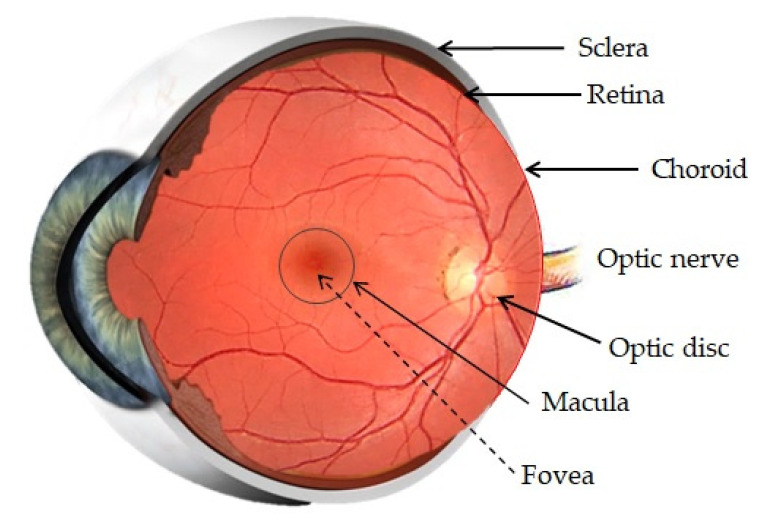
Anatomy of the posterior segment of the eye. Adapted from [24,25] Akbar, S., 2017 and Bharali, P., 2015.

The posterior segment is formed by three main layers, the sclera, choroids and retina, all around the vitreous cavity, containing the vitreous humor (Figure 2). Penetration of drugs or other foreign substances into the posterior segment is restricted by the BRB, located between the choroid and the retina [19].

##### Sclera

The sclera is a strong external coat formed by connective tissue (such as collagen, elastic fibers or fibroblasts). The sclera maintains the shape of the eye, contributes to the preservation of intraocular pressure (IOP), provides points of attachment for extraocular muscles and has a protective function [19,20].

##### Choroid

The choroid is a vascular and innervated layer which nourishes the posterior eye. It is formed by fenestrated capillaries and supported by Brunch’s membrane, which is the basement membrane of the RPE and regulates the exchange of metabolites and nutrients between the choroid and the RPE [19,20]. Lipophilic substances can permeate across the choroid and present poor permeability to proteins and small hydrophilic compounds. Drugs can be transported by the transcellular route and the paracellular route. The transport of the majority of the drugs is achieved by both pathways [21,22].

##### Vitreous Humor

The vitreous humor is a transparent gel composed of various collagen types and glycosaminoglycans dissolved in water. It contributes to maintenance of the ocular structure and is involved in nutrient transport. When a drug arrives to the vitreous humor, it can move through diffusion because the vitreous does not offer diffusional resistance to movement of small and anionic particles, or it can move through convection in elder population due to the fact that the vitreous becomes more liquid with ageing [18,19,20].

##### Blood Retinal Barrier

The BRB is divided into two parts: the outer and the inner BRB. The outer barrier is composed by the RPE and the Bruch’s membrane, and its function is to regulate the access of nutrients from the choroid to the subretinal space. The inner barrier lines the retinal vasculature and protects the retina from foreign substances in the blood circulation. Both are formed by tight junctions, which form a selective obstacle that restricts penetration of molecules into the intraocular chamber [10,19,20].

##### Retina

The retina is the neurosensory inner coat of the posterior segment of the eye, which encloses the inner surface of the posterior segment except at the optic disc, where the optical nerve is located, and ends in the ora serrata, next to the pars plana ciliary epithelium. The retina is formed by some layers of the RPE, which aid in maintaining photoreceptor function, retinal adhesion and in the production of growth factors. This RPE layers are separated from the neural retina by the subretinal space. Two types of photoreceptors exist, rods and cones, which are adjacent to the RPE, and their distribution varies in different regions of the retina [19,20]. The RPE is a single-layered structure with tight junctions which is outside the neurosensory retina. This pigment participates in drug uptake into the retina and vitreous humor after trans-scleral or systemic administration of drugs. Hydrophobic drugs with low molecular weight can be absorbed by the choroid-RPE system [13,23].

### 2.3. Drug Delivery Routes

**Figure 3:**
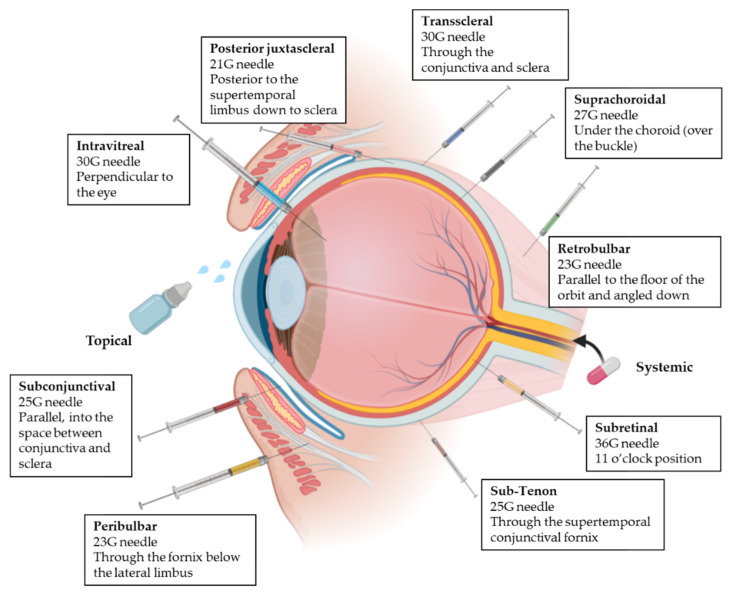
Representation of the different delivery routes for ocular administration [31,32]. Next to each administration, needle and injection technique are summarized. Adapted from [23,33,34,35,36,37,38,39], Varela-Fernandez, R., 2020; Doshi, R., 2011; Moshfeghi, D.M., 2002; Yïu, G., 2020; El Raves, E.N., 2013; Do, J.L., 2020; Pakravan, M., 2017; Davis, J.L., 2019.

All the mentioned ocular barriers constitute a challenge for drug delivery to the posterior segment of the eye. For this reason, besides topical administration, different administration routes can be applied to ensure that drug concentrations are maximized at the target site overcoming some of the ocular layers [3,17]. This following section introduces the four ocular drug administration routes most widely used to achieve the posterior segment in clinical practice (Figure 3) [26,27].

#### 2.3.1. Topical Administration

Topical administration is the most patient friendly ocular drug delivery method. The eye drop application ensures significant drug concentration in the anterior segment, but it is usually insufficient to achieve a therapeutic concentration in the posterior segment due to the anatomy and physiology of the eye. Penetration of drugs can be achieved using several mechanisms:

1. Diffusion into the iris and, consequently, into the aqueous humor of the posterior chamber and innermost tissues.
2. Permeation through the pars plana in the ciliary body, without crossing the BRB.
3. Lateral diffusion across the sclera followed by penetration of Bruch’s membrane until the RPE.
4. Absorption by systemic circulation through the conjunctival vessels or via nasolacrimal duct, and then the drug can access to the retinal vessels.

In addition, the mechanism and magnitude of drug penetration and tissue distribution depend on the physicochemical properties of the molecule [20,28,29,30].

In this area, C. Pugliaet al. studied ocular distribution of marked lipid nanoparticles [40]. They synthesized fluorescently labeled lipid nanoparticles and applied them topically to a mice preclinical model. Animals were sacrificed after 1, 3, 8 and 16 h after the administration. Their results showed that the diffusion of nanoparticles began at the corneal level and spread towards the back of the eye to reach the sclera and the retina. Moreover, they hypothesized that scleral absorption was one of the main routes due to the absence of fluorescent signal in the aqueous body, lens and vitreous body.

#### 2.3.2. Systemic Administration

Systemic administration is based on drug delivery by conventional routes (oral, intravenous, or intramuscular), where the drug reaches the bloodstream. It allows us to deliver drugs into the choroid due to its vascularization. However, the penetration of the drugs into the posterior segment is limited by the BRB, which controls the entry of drugs from the choroid into the retina. In addition, other limitations are the possible degradation of the drug before reaching the ocular target and the systemic side effects. By systemic administration, only between 1 and 2% of the administrated drug can access to the retina and vitreous body [10,11,20,41].

In this area, M. Occhiutto et al. studied the effects of placitaxel lipid nanoparticles administered intravenously after a trabeculectomy [42]. They compared four groups of rabbits, mitomycin-C suspension (MMC) (antifibrotic), lipid nanoparticles subconjunctivaly injected (LP-SC), lipid nanoparticles intravenously injected (LP-IV) and a control group. In the LP-IV group, they found out that there was not significant toxicity, meaning that lipid nanoparticles avoided the adverse effects of the drug. However, LP-IV antiscarring therapeutic action was slightly inferior in comparison to MMC and LP-SC. Therefore, intravenous injection of nanoparticles was able to decrease the formation of fibrous tissue in the subconjunctival space and to increase the bleb survival, but subconjuctival routes effects were increased. They concluded the study hypothesizing that LP-IV could be used as an adjuvant in the wound-healing modulation in trabeculectomy.

#### 2.3.3. Intravitreal Administration

In the intravitreal route the drug is administrated by direct injection into the vitreous humor. It achieves high doses of active compounds in the posterior segment compared to other routes and also minimizes systemic side effects. The composition of the vitreous humor affects the drug penetration and distribution. The distribution of the drug depends on the molecular weight and pathophysiological condition of the vitreous humor. Additionally, increased particle size decreases the mobility in the vitreos humor [43]. Furthermore, some components of the vitreous humor, such as collagen, glycosaminoglycans and hyaluronan, have negative charge, leading to the interaction with the cationic particles restricting their movement. Nevertheless, if repeated injections are used, it can lead to ocular side effects, such as retinal detachment, iritis, uveitis, endophthalmitis and intraocular hemorrhage. In addition, other side effects are pain and discomfort, which induces patient noncompliance [3,10,20].

In this area, intravitreal injection was used by X. Huang et al. to investigate the accumulation and distribution of differently charged lipid nanoparticles after intravitreal administration in mice [44]. At the pars plana, 1 μL was administered, and the studied lipid nanoparticles were either negative, neutral, mildly positive or highly positively charged. The results showed that negative, neutral and mildly positive particles were cleared out fast, with a half-life around 3 h; meanwhile, the positive ones had a half-life of 27 h. These cationic lipid nanoparticles also achieved the inner tissues (retina and RPE).

#### 2.3.4. Periocular Administration

Periocular delivery includes subconjunctival, retrobulbar, peribulbar, sub-Tenon, suprachoroidal, trans-scleral, subretinal and posterior juxtascleral route. Whereas peribulbar, posterior juxtascleral, sub-Tenon and retrobulbar routes are usually used for administration of anesthesics [26,32], subconjunctival, suprachoroidal, subretinal and trans-scleral routes are used for delivering active compounds to inner tissues; (i) the subconjunctival route involves the insertion of the drug or implant underneath the conjunctiva, which allows the access to the sclera [26,32,45]; (ii) the suprachoroidal route is used for implants, microneedles and other formulations. The drug is administered between the sclera and choroid, minimizing the systemic side effects [20,46]; (iii) the subretinal route involves the injection directly into the posterior ocular segment and subsequently diffuses to the RPE layer and the inner retina [47]; (iv) the trans-scleral route allows drugs to permeate through inner ocular tissues to reach the neuroretina. Implant and iontophoretic devices are used, which are less invasive and safer than intravitreous administration [3,46].

Inthis area, subretinal administration, was investigated by D. Delgado et al. who prepared SLNs and administered them through three different routes: intravitreal, subretinal and topical [48]. The results showed that there was a good response in retinal ganglion cells when intravitreal injection was employed, but protein expression was poor in RPE cells. Otherwise, after subretinal injection, the vectors were able to transfect RPE cells as well as photoreceptors. After topical application, the vectors were also able to transfect corneal cells.

## 3. Lipid Nanoparticles

Achieving therapeutical doses in the eye with topical ocular drugs is highly difficult due the ocular anatomy and physiology. This obstacle has led to the development of new drug delivery systems which carry out effective drug concentrations in the inner tissues and prolong the time of residence [49,50]. The penetration through the ocular barriers can be possible by the employment of small particles, such as polymeric nanoparticles, liposomes, hydrogels, nano-micelles, dendrimers, and nanosuspensions [23,51].

Polymeric nanoparticles are solid colloidal systems in which the active compound is encapsulated onto the constituent polymer matrix [52]. Polymeric nanoparticles provide several advantages for ocular delivery, such as biodegradability, nontoxicity, biocompatibility and mucoadhesiveness [53]. However, the problems with these polymeric nanoparticles are the cytotoxic potency of the formulation and the cost-effective manufacturing procedures on a large scale [7].

On the other hand, liposomes are spherical vesicles consisting of one or more concentric phospholipid bilayers. Liposomes are able to enhance the active corneal permeability because of their ability to come in close contact with cornea and conjunctiva as well as increase the extent of corneal uptake by prolonging the corneal contact time [54,55]. Otherwise, liposomes have chemical and physical stability problems due to the aggregation and drug degradation [56].

Other drug delivery systems such as hydrogels have been used in ophtalmic applications due to their ability to prolong residence time and muchoadhesive properties. Hydrogels are three-dimensional polymers with a high water-absorption capability and excellent biocompatibility. However, hydrogels function depends on temperature, pH or ion trigger [57,58].

Nanosuspensions are very finely colloid, biphasic, dispersed, and solid drug particles in an aqueous vehicle. Nanosuspensions also provide a prolonged contact time of the formulation with the ocular surface, but they could cause blurred vision [57,59].

Nanomicelles, on the other hand, encapsulate nanosized hydrophobic drug molecules into a colloidal drug delivery system formed by block or amphiphilic polymers. The problem with nanomicelles is that the drug loaded encounters rapid tear dilution upon their topical administration, obtaining a premature drug release. In addition, nanomicelles are costly compared to other formulations [60,61]

In 1990, M.R. Gasco and R.H. Müller developed an alternative to the mentioned nanosystems, lipid nanoparticles, which consist of a lipophilic matrix of particle size between 100 and 1000 nm and an aqueous phase. The first generation of lipid nanoparticles were named solid lipid nanoparticles (SLNs). Later, in 1999, the second generation of lipid nanoparticles was developed, namely nanostructured lipid carriers (NLCs) [62].

### 3.1. Types of Lipid Nanoparticles

As mentioned above, two types of lipid nanoparticles exist: SLNs and NLCs. Both share some characteristics, such as their biocompatible and biodegradable composition, the feasibility of encapsulating hydrophilic and hydrophobic substances and the ease of large scale production [63,64].

#### 3.1.1. Solid Lipid Nanoparticles

SLNs are made from lipids that are solid at body temperature, such as fatty acids, fatty alcohols, glycerol esters and waxes. Furthermore, an SLN formulation includes stabilizing agents, mainly surfactants, which reduce the interfacial energy between the lipid phase and the aqueous phase during the preparation of the nanoparticles. The active compound is usually added in the lipid phase due to the fact that most drugs have low solubility in water. However, hydrophilic drugs can also be added to be encapsulated using different preparation methods [63,64,65].

**Figure 4:**
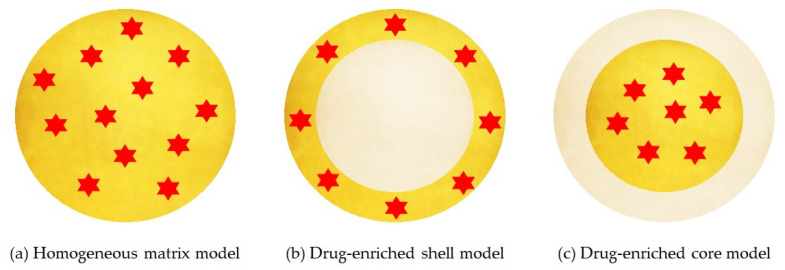
Three models of SLN drug incorporation: (**a**) homogeneous matrix model, (**b**) drug-enriched shell model, (**c**) drug enriched core model. Red color indicates the active compound encapsulated, yellow and grey color refer to lipid phases with and without dispersed drug, respectively. Adapted from [63], Müller, R. H., 2000.

The location of the drug depends on the structural organization of the colloidal lipid system, and this structure varies according to the formulation composition and the production method. There are three different models for the incorporation of the drug into SLNs, represented in Figure 4:

- Homogeneous matrix model: the drug is distributed homogeneously around the structure of the nanoparticle. This model is mainly obtained by high pressure homogenization due to the fact that the drug is dissolved in the bulk lipid and, through mechanical breaking, leads to nanoparticles with homogeneous matrix structure [66].
- Drug-enriched shell model: the drug is concentrated on the particle surface. This model can be obtained when there is a phase separation during the cooling process and the lipid precipitates on the surface and crystallises. This type of SLN allows a fast release [66].
- Drug-enriched core model: the drug is concentrated inside and is surrounded by the lipid matrix. This model occurs when the drug precipitates in the centre of the particle [66].

On the other hand, SLNs have some potential problems mainly due to their stability during storage. When SLNs crystallise during the preparation method, they form a higher energy modifications α and β’. During storage, these two modifications can transform to a more ordered β modification, promoting the expulsion of the drug. Other disadvantages of the SLNs are the low payload of the drug and the high-water content [64,66].

#### 3.1.2. Nanostructured Lipid Carriers

In order to solve the instability problems of the SLNs, the second generation of lipid nanoparticles was developed, namely NLCs. In these particles, the matrix is composed of a solid lipid and a liquid lipid (e.g., oil). The advantages of NLCs over SLNs are mainly a better loading capacity of drugs due to its imperfect structure and its improved stability because they do not allow the recrystallization of the solid lipid [62,66,67].

**Figure 5:**
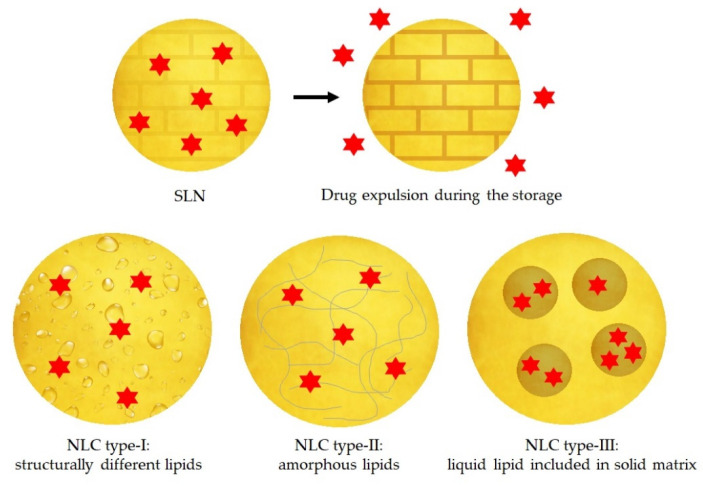
Representation of the drug expulsion during storage of SLNs and three types of NLC: type-I structurally different lipids, type-II amorphous lipids and type-III liquid lipid included in solid matrix. Red color indicates the active compound. Adapted from [68,69], Müller, R.H., 2002; Narvekar, M., 2014.

Depending on the production methods and the composition of the lipid matrix, there are three different types of NLC, represented in the Figure 5:

- The imperfect type: it is formed when a small amount of liquid lipid is mixed with the solid lipid, resulting in a less perfect crystalline structure [63].
- The amorphous type: it is formed when solid and liquid lipids are mixed as well, and then the lipid matrix is solid but not crystalline. It can be achieved using characteristic lipids, such as hydroxyoctacosanylhydroxystearate with isopropylmyristate [63,66].
- The multiple type: it is a multiple system where the solid lipid matrix contains small liquid oil nanocompartments. It is formed when the amount of liquid lipid is increased, and it is not able to be completely incorporated into the crystal lattice. When the liquid lipid concentration increases, the solubility of the oil molecules in the solid lipid is exceeded, and a phase separation is produced promoting the oily nanocompartments. This type of NLC is formed by hot homogenization method during the cooling process [63,66].

### 3.2. Preparation Methods of Lipid Nanoparticles

Different preparation methods have been developed for the obtention of lipid nanoparticles. The physicochemical properties and the stability of the drug and lipid nanoparticles are influenced by the preparation method. For this reason, the choice of the preparation method is remarkably important [70]. In the following section, the preparation methods found in the literature are summarized.

#### 3.2.1. High-Pressure Homogenization

The high-pressure homogenization is a well-established and powerful technique. It consists of pushing a liquid with high pressure through a narrow gap. The fluid accelerates trough a very short distance at very high velocity, leading to shear stress and cavitation forces that shatter the particles down to the submicron range [56]. There are two different procedures to perform this technique: hot and cold homogenization.

- Hot homogenization: the procedure is carried out at temperatures higher than the melting point of the solid lipid. The drug is melted with the lipids and then dispersed in a hot aqueous phase with surfactants by a high-shear mixing device [56,71]. Afterwards the system is cooled down and the lipid solidifies, forming the nanoparticles.
- Cold homogenization: the drug is solved in the lipid melted mixture and is quickly solidified by cooling down with dry ice or liquid nitrogen. This solid phase is then grounded into a fine powder by milling it into microparticles. These are dispersed in a cold aqueous phase with the surfactants [56,71].

#### 3.2.2. Solvent Emulsification-Evaporation

The lipid is dissolved in a water-immiscible organic solvent and is emulsified in an aqueous phase by high speed homogenization. Afterwards, the solvent is evaporated by mechanical stirring at room temperature and reduced pressure, forming the lipid nanoparticles [71,72].

#### 3.2.3. Solvent Emulsification-Diffusion

In this method, a water-miscible solvent and water are saturated with each other. The lipid and the drug are dissolved in water saturated solvent, and this organic phase will be later emulsified with solvent saturated aqueous solution containing stabilizer using mechanical stirring. Then, water is added to the emulsion and the solvent is eliminated by vacuum distillation or lyophilization [71,72].

#### 3.2.4. Ultrasonication or High Shear Homogenization

The procedure is carried out at temperatures higher than the melting point of the solid lipid. The lipid is melted, dispersed into the warm aqueous phase, which contains surfactant, and then is emulsified by probe sonication or by high-speed stirring. The pre-emulsion is placed into ice-water bath and ultrasonicated using probe sonicator [63,70,71].

#### 3.2.5. Supercritical Fluid Extraction of Emulsions

The lipid material, the drug and the surfactant are dispersed into an aqueous solution, and then the mixture is introduced in a high-pressure homogenizer to form an emulsion. Afterwards, the mixture is introduced from one end of the extraction column at a constant flow rate, and the supercritical fluid is introduced at a constant flow rate in a counter-current manner. Finally, lipid nanoparticles are obtained by continuous extraction of solvent from the emulsion [72,73].

#### 3.2.6. Microemulsion Based Method

A mixture of a low melting fatty acid, an emulsifier and water are stirred at a temperature higher than the melting point of the lipid. The hot microemulsion is dispersed in cold water under stirring [70,71].

#### 3.2.7. Double Emulsion Method

The aqueous phase containing the drug is added to the melted lipid and surfactant at a temperature higher than the melting point of the lipid. Then, this microemulsion is added to a mixture of water and surfactant to obtain a w/o/w system. The nanoparticles can be obtained by dispersing the emulsion in cold water and then washing with dispersion medium by an ultrafiltration system [67,74].

#### 3.2.8. Precipitation Method

The lipids and the drug are dissolved in an organic solvent, and then it is emulsified in an aqueous phase. The organic solvent is evaporated and the lipid precipitates, forming lipid nanoparticles [63,67].

#### 3.2.9. Film-Ultrasound Dispersion

The lipid and the drug are mixed into an organic solvent, which is evaporated, and a lipid film is formed. Afterwards, the aqueous solution with the surfactant are added to the film. Using the ultrasound probe to diffuse, the lipid nanoparticles are formed [56,71].

#### 3.2.10. Solvent Injection Method

The lipid is dissolved in a water miscible solvent; then it is injected through an injection needle into a stirring aqueous phase containing a surfactant. The dispersion is filtered to remove any excess of lipid [70].

#### 3.2.11. Membrane Contractor Method

The procedure is carried out at temperatures above the melting point of the solid lipid. The mixture is pressed through the cylindrical membrane pores, allowing the formation of small droplets. Inside the membrane module, the aqueous phase circulates and sweeps away the droplets forming at the pore outlets. Lipid nanoparticles are formed by the subsequent cooling of the mixture to room temperature [67,70].

#### 3.2.12. Phase Inversion Temperature Method

All the components are mixed under constant magnetic stirring. The mixture is subjected to three cycles of heating and cooling. Finally, cold water is added to the emulsion, causing phase inversion of the emulsion and breaking, resulting in the formation of lipid nanoparticles [67,70].

#### 3.2.13. Coacervation Method

The drug is solubilized in ethanol and then is added to a solution prepared previously with the lipid, with constant stirring until a single phase is formed. A suspension is yielded by the gradual addition of a coacervating solution to this mixture. Cooling the suspension in a water bath under agitation promotes the formation of the lipid nanoparticles [67,70].

#### 3.2.14. Particles from Gas Saturated Solution (PGSS) Method

Supercritical CO_2_ and the melted lipid are mixed with the drug, and then a fast expansion through a nozzle is produced, causing the atomization of the melted lipid, the complete evaporation of the gas and the precipitation of the lipid nanoparticles [75].

## 4. Physicochemical Properties of Lipid Nanoparticles for Drug Delivery to the Posterior Segment

Lipid nanoparticles provide new strategies to overcome the limitations of the conventional dosage forms. Nanotechnology is able to increase the solubility of drugs, avoid metabolic degradation, decrease dosing frequency and make drug targeting possible [76]. Despite the potential of lipid nanoparticles for ocular drug delivery, there are not official guidelines about lipid nanoparticles characteristics for ocular administration. In the following sections, the pre-clinical studies and the information about these nanoparticles are summarized.

### 4.1. Physicochemical Characteristics of Nanoparticles to Achieve Posterior Segment

Physicochemical properties of the nanoparticle are highly important parameters to be considered when developing pharmaceutic products. Particle size distribution, polydispersity index (PDI) and zeta potential (ZP) can affect the bulk properties, product performance, processability, stability, and appearance of the ended product [77].

Generally, particles ≤ 200 nm are considered to offer reasonable permeation and mobility through the ocular barriers. However, particles < 20 nm are quickly cleared out. Values of PDI < 0.3 generally indicate a homogeneous distribution of nanoparticles. Moreover, zeta potential is related to the stability of the nanosystem. High absolute values, about ± 20 mV, overcome the attractive forces between particles, achieving a better stability of the colloidal system. In addition, if the topical route is used for the administration of the nanoparticles, a cationic charge promotes electrostatic interactions between the surface of the cationic particles and the anionic ocular mucosa, with a considerable improvement of the drug residence time. Finally, a high entrapment efficiency (EE) is desirable to attain the maximum drug amount inside the nanoparticle [78,79,80].

### 4.2. Excipients for Ocular Drug Delivery

The eye is a sensitive organ, which means that toxicity of every component of the formulations must be studied. Excipients play an important role in lipid nanoparticles because they could confer additional properties to the formulations, such as the control of the release rate of the drug. Excipients for ocular delivery should (i) accomplish safety with no local and systemic side effects, (ii) increase the ocular residence time of the drug administered, (iii) control drug release, (iv) be stable and easy to handle, (v) be compatible with the drug, and (vi) be biodegradable and biocompatible. Excipients in ophthalmic drug delivery systems can be classified according to their function in the drug delivery systems [81,82]. In the following sections, excipients for lipid nanoparticles are classified.

#### 4.2.1. Lipids

Lipid nanoparticles are formed by solid lipids (SLNs) and liquid lipids (NLCs), which are physiological, biodegradable and nontoxic [83,84]. Lipids have been approved by the European and United States regulatory authorities for ocular applications, and they are Generally Regarded as Safe (GRAS) [84,85].

##### Solid Lipids

Solid lipids are lipids which form a highly ordered crystalline lattice. They are solid at body temperature, which allows a controlled and sustained drug release [14]. Solid lipids are the main component of the formulation in lipid nanoparticles. For this reason, the decision of which solid lipid to use is highly relevant, and lipid screening is used as a tool to choose the lipid that has a better solubility with the active compound. However, there are no standard methods for determining the solubility of a drug molecule in a solid lipid excipient [86,87]. An example of a method to determine the best solubility of a solid lipid with a drug is the used by A. Kovačević [88]. They added an amount of the active compound to a different solid lipids, and they melted it. The mixtures were cooled down for 24 h and analyzed by light microscope. After solidification, the mixture was inspected for the presence of drug crystals by light microscopy, and the lipid chosen was the one with fewer drug crystals.

Solid lipids used in the preparation of lipid nanoparticles for ocular drug delivery are Compritol^®^ 888 ATO [89], Precirol^®^ ATO [90], glyceryl monostearate [91], Gelucire^®^ 44/14 [92], Phospholipon^®^ 90G [93], stearylamine [94], Dynasan^®^ 116 [95], stearic acid [96], Softisan^®^ 100 [97].

##### Liquid Lipids

Liquid lipids are incorporated in the NLCs in order to overcome disadvantages of SLNs. Liquid lipid influences physicochemical properties of nanoparticles, such as particle size, viscosity, and drug distribution. A few liquid lipids are biodegradable and nontoxic [98]. There are no standard methods to determine the highest solubility between liquid lipid and the drug. However, one of the most widely used screening methods for liquid lipids is the described by P. Sathe et al. [99]. They studied the maximum solubility of the active compound by HPLC. They mixed the drug with several liquid lipids and incubated them for 24 h. The mixtures were centrifugated and the supernatant was diluted to quantify the active compound. The mixture which contained a higher amount of drug was considered the most suitable for drug solubilization.

Some of the most widely used liquid lipids for ocular drug delivery are Lutrol^®^ F68 [100], Miglyol^®^ 812 [101], castor oil [102], and oleic acid [103].

#### 4.2.2. Penetration Enhancers

Penetration enhancers allow the nanoparticle to penetrate the cornea and decrease barriers resistance. These excipients increase the permeability of the ocular tissues temporarily and allow nanoparticles—and, consequently, the drug—to pass through ocular tissues. Surfactants are the most used penetration enhancers in lipid nanoparticles preparation. In addition, they play an important role in the physical stability of the nanoparticle and drug permeability into ocular cells [104]. Cyclodextrins can be also used as penetration enhancers, but they have not been extensively used in lipid nanoparticles. Moreover, the lipids of the matrix can also act as penetration enhancers [82,105].

##### Cyclodextrins

Cyclodextrins are water-soluble cyclic oligosaccharides. They have lipophilic cavities where the active compound can reside; meanwhile, it is protected but not covalently bound. However, cyclodextrins are large molecules; they cannot permeate through lipophilic membranes, such as the corneal epithelium. For this reason, F. Wang et al. synthesized nanoliposomes encapsulating a complex of brinzolamide and an hydropropyl-β-cyclodextrin [105,106]. With this novel strategy, the presence of cyclodextrin in the aqueous compartment of nanoliposomes would not affect the characteristics of conventional liposomes but prolong drug release compared to conventional liposomes. The formulation was prepared in order to improve local brinzolamide glaucomatous therapeutic effect. They obtained nanoliposomes with a particle size of 80 nm, PDI of 0.21 and a ZP almost neutral, about −3 mV. The entrapment efficiency of the formulation was high; more than 90% of the drug was encapsulated. Furthermore, they studied the corneal permeation, and they obtained a sustained release of the active compound. Finally, they tested their formulation in an in vivo model of glaucoma. The results showed that in 1 h after the administration of the novel formulation, the IOP decreased and maintained for 12 h, even the dosage of brinzolamide was just 10% compared to the commercially available formulation. Therefore, this strategy may also be useful for lipid nanoparticles.

##### Surfactants

Surfactants are substances that reduce the surface tension. As it is mentioned above, surfactants used in preparation of lipid nanoparticles have influence on the physical stability, drug permeability, and also, they can contribute to the safety of lipid nanoparticles when administered to the body [104,105]. Three types of surfactants can be incorporated into lipid nanoparticles, and these can be classified in terms of their charge: cationic, anionic, and non-ionic.

- Cationic surfactants—they have a positive charge on the polar head group. Some of the cationic surfactants used for lipid nanoparticles are the following: cetylpyridinium chroride [105], cetyltrimethylammonium bromide [107], dimethyldioctadecylammonium bromide [79], octadecylamine [95], and benzalkonium chloride [108]. However, at high concentrations, they can cause ocular irritation.
- Anionic surfactants—they have a negative charge, but they are not recommended for ocular drug delivery because they can cause ocular irritation [109].
- Non-ionic—they have neutral charge. Non-ionic surfactants are the compounds of choice for ocular drug delivery, bringing enhanced drug solubility, formulation stability, biocompatibility, and low toxicity compared with cationic and anionic surfactants [105]. The most used non-ionic surfactants are polysorbate 80 [110], poloxamer 188 and 407 [111], and sorbitane monostearate 60 [112].

Other surfactants used for ocular delivery are Transcutol^®^ and Labrasol^®^ because of their ability to enhance corneal penetration [113,114].

##### Fatty Acids

Fatty acids are able to enhance ocular drug permeation by altering cell-membrane properties and loosening tight junctions. Caprylic acid and capric acids are examples of penetration enhancers [105,115]. As an example of lipid matrix formed by capric acid, Chi-Hsien Liu et al. prepared two formulations of lutein loaded NLCs to study the corneal distribution, in order to treat macular degeneration [112]. One formulation contained cyclodextrins (NLC-D). NLC-D formulation was bigger than the naked NLC (360 and 190 nm, respectively), but the corneal accumulation and partition coefficient of lutein were improved by NLC-D. Therefore, the addition of cyclodextrins enhanced the viability of corneal cells.

#### 4.2.3. Viscosity-Enhancing Agents

Viscosity enhancers improve precorneal residence time and bioavailability upon topical drop administration by enhancing formulation viscosity. Gels are commonly used for the preparations due to their high viscosity. Classical gels contain excipients which make the formulations viscous, and they can be directly applied to the ocular surface. However, as a disadvantage, they can result in blurred vision during the application. Moreover, it is difficult to administer an exact dose of the gel due to the high viscosity. In order to improve this issue, there are other viscosity enhancers that need exposure to specific physiological conditions to increase their viscosity, such as temperature, pH, or ion concentration. In the presence of these stimuli, they increase their viscosity forming in situ gels [82,116]. However, some in situ gels have some disadvantages due to the risk of gelling before administration, such as thermally-responsive gels [82]. In this area, A. Tatke et al. prepared triamcinolone acetonide loaded SLNs with gellan gum, which is a polysaccharide that forms a gel in contact with the ions present in the tear film of the eye [117]. The formation of the gel is due to the presence of cations that causes the cross-link of the polymer. The study showed that the formulation provided higher drug concentration in tear and in the anterior and posterior segments compared to water-dispersed SLNs. Therefore, in situ gel enhanced active compound penetration.

Additional examples of excipients for the formation of gels are hydroxy methyl cellulose, hydroxy ethyl cellulose, sodium carboxy methyl cellulose, hydroxypropyl methyl cellulose, and polyalcohol [116].

#### 4.2.4. Bioadhesives/Mucoadhesives

To improve retention time at the corneal surface and improve corneal permeation through endocytic uptake by cornea epithelial cells, excipients with adhesive properties can be used. Cationic lipids or bioadhesive polymers can be added into the formulation for this purpose [82,118].

##### Cationic Lipids

Cationic lipids provide a positive surface charge to the nanoparticles, leading to an electrostatic attraction between the particle and the negative surface of ocular mucosa. This approach increase drug retention time in the eye, improving nanoparticles bioadhesion [97,107].

##### Bioadhesive Polymers

These polymers can be associated with lipid nanoparticles to improve the residence time of the particles in the precorneal area, enhancing drug penetration across epithelia [82,119]. Most widely used polymers are hydroxypropyl methyl cellulose [120], polyvinyl alcohol [121], sodium hyaluronate [122], chitosan [123]. As an example of application of these systems, F. Wang et al. prepared SLNs loaded methazolamide coated with chitosan for the treatment of glaucoma [124]. The results showed that the combination of SLNs with chitosan, conferred a positive surface charge and higher bio-adhesivity, improving the retention time of the formulation. Furthermore, they compared the in vivo efficacy of their novel formulation against commercial methazolamide eye drop and SLNs without chitosan. The results of the assay showed a sustained and longer antiglaucomatous effect of the chitosan coated SLNs, indicating the favorable properties of the novel formulation.

#### 4.2.5. Other Excipients

There are other excipients that can be added into the formulation to offer prevention against microbial growth (preservatives), against undesirable physical/chemical reactions, maintenance of pH, enhancement of stability, or cryoprotection of the formula [82]. These excipients are used also in the conventional formulations and they are approved by regulatory administrations such as FDA and EMA.

## 5. Lipid Nanoparticles for the Posterior Segment

Posterior ocular segment diseases are one of the principal causes of irreversible blindness worldwide, mainly in the aging population [125,126]. In this sense, lipid nanoparticles constitute an interesting drug delivery system due their unique characteristics, making it possible to achieve therapeutical doses in the inner tissues of the eye. They are made from lipids, either from natural or synthetic sources, thus avoiding acute and chronic toxicity. In addition, they present mucoadhesive properties, which offers an enhancement of the residence time of the drug increasing its ocular bioavailability, and they can be sterilized [51,71,127,128].

In the following sections, the most common diseases that affect the posterior segment of the eye, its pharmacological treatment and lipid nanoparticles designed to treat them are summarized.

### 5.1. Age-Related Macular Degeneration (AMD)

AMD is a disease that affects the macular region of the retina, characterized by a progressive visual impairment due to neurodegeneration of the photoreceptors and RPE. Usually, the first clinical finding of AMD by funduscopic examination is the presence of drusen, which are focal deposits of acellular and polymorphous extruded material between the RPE and Brunch’s membrane that appear with ageing. AMD can be divided in two main types of macular degeneration [49,129]:

- Dry AMD, also known as the non-exudative form, is the most common type [49,129].
- Wet AMD is the exudative form, associated with a fastest progression causing a quick vision loss [49,129].

Dry AMD has no approved pharmacological treatment, whereas wet AMD is treated pharmacologically, but none of the currently used drugs are able to cure the disease or reverse its course. Nowadays, the most common and effective clinical treatment for wet AMD is anti-VEGF (Vascular Endothelial Growth Factor) therapy, which implicates periodic intravitreal injections. The first anti-VEGF agent was pegaptanib, which was substituted for newer and more effective anti-VEGF agents, as ranibizumab and bevacizumab, two recombinant humanized monoclonal IgG1 intravitreal administered. Aflibercept is the latest approved anti-VEGF agent, a VEGF-A receptor decoy [129,130,131]. Novel treatment approaches using lipid nanoparticles are summarized in Table 1.

In this sense, M. Yadav et al. designed atorvastatin-loaded SLNs for the treatment of AMD [132]. Some clinical and epidemiological studies have shown that cardiovascular disease and AMD might have similar ethiology due to their shared risk factors, such as aging changes in some ocular structures, and genetic predisposition. The Brunch’s membrane lining lying below the RPE forms the inner margin of the choriocapillaries, which is considered a structural analogue of the vascular intima of arteries. Furthermore, there is some modification, oxidation and aggregation of some proteins of the eye. For these reasons, statins, as atorvastatin, might constitute a therapeutic approach for AMD. Yadav et al. fabricated SLNs by hot high pressure homogenization for topical administration [132]. The atorvastatin-loaded SLNs were able to provide enhanced permeation across cornea and a high bioavailability in aqueous and vitreous humour. The lipid nanoparticles showed safety in vitro with corneal and retinal cell lines and in vivo with acute and subchronic toxicity studies.

Other developing treatment of dry AMD is the administration of pentamethyl-6-chromanol, a potent antioxidant which has protective effects for RPE. A. Arta and coworkers designed SLNs loading pentamethyl-6-chromanol prepared by high-shear homogenization [133]. They tested in vitro the protective function and observed the suppression of reactive oxygen species (ROS) and the prevention of the accumulation and destabilization of other oxidants molecules, as lysosomal ox-LDL.

Regardless of the investigations of lipid nanoparticles for the treatment of AMD, there are no clinical trials of SLNs or NLCs. The system BioSeizer^®^, which is based on a multilayer lipid membrane developed by TLC, is a drug delivery system similar to lipid nanoparticles currently on clinical trials. In the phase II clinical trial NCT02006147, they are testing the efficacy and tolerability of TLC399, which encapsulate dexamethasone for the treatment of macular edema by intravitreal injection every six months [134,135].

### 5.2. Diabetic Retinopathy

Diabetic retinopathy is the most common microvascular complication of diabetes mellitus. Moreover, vision loss occurs in advanced stages of the disease, called diabetic macular edema (DME) and proliferative diabetic retinopathy (PDR). Generally, it advances from mild nonproliferative abnormalities, where the number of microaneurysms increases, until moderate and severe stages, where new blood vessels grow in the retina and posterior surface of the vitreous [136,137].

The pharmacological treatment of diabetic retinopathy is based on the intravitreal injection of ranibizumab, bevacizumab or aflibercept. Otherwise, for more than 40 years the main treatment for diabetic retinopathy has been the laser (precision photocoagulation), used to eliminate discrete vascular abnormalities in the retina [136,137,138]. New treatment approaches using lipid nanoparticles are summarized in Table 1.

A novel treatment for diabetic retinopathy is SLNs containing siRNA silencing HuR expression [139]. M. Amadio and colleagues demonstrated that HuR protein is upregulated and it binds to the VEGF-encoding mRNA, which promotes the abnormal increase of VEGF in the retina. For this reason, they fabricated SLNs by quasi-emulsion solvent diffusion. The research group observed that animals treated with naked siRNA showed no significant retinal protection after intravitreal injection. Instead, the coated-SLNs significantly reduced HuR and VEGF.

### 5.3. Uveitis

Uveitis is the infectious or non-infectious inflammation of the uveal tract and adjacent structures of the eye, depending on the anatomic site of the maximum inflammation. When it affects the choroids or the retina, it is called posterior uveitis, one of the most severe forms. In the treatment of the posterior uveitis, intravitreal injections of corticoids are used (triamcinolone acetonide and dexamethasone phosphate). Furthermore, there are two fluocinolone acetonide intravitreal implants, and a biodegradable dexamethasone drug delivery system of poly(lactic-co-glycolic acid) (PLGA) approved [140,141,142]. As a biological therapy, efalizumab, a humanized monoclonal antibody against CD11, is used in a complication of the posterior uveitis, the macular edema. If there is neovascularization, anti-VEGF can be used, such as pegaptanib, ranibizumab and bevacizumab [94,143,144]. New treatment approaches using lipid nanoparticles are summarized in Table 1.

A new specific treatment for uveitis designed using lipid nanoparticles is NLCs loading triamcinolone acetonide [94]. P. Nirbhavane et al. designed these nanoparticles which showed a slow and sustained in vitro release and sustained ex vivo transcorneal permeation. They confirmed that the formulation possessed anti-inflammatory activity.

### 5.4. Glaucoma

Glaucoma is a group of ocular diseases characterized by the neurodegeneration of the retinal ganglion cell axons and somas, leading to a loss of vision [145]. It is one of the main causes of blindness worldwide, with 6.9 million of patients affected. The major risk factor for developing glaucoma is elevated IOP. There are 11 varieties of glaucoma, but 90% of all glaucoma cases have a high IOP, categorized as primary open-angle glaucoma. The pharmacological treatment of glaucoma initiates with monotherapy of antiglaucoma drugs, such as prostaglandins (latanoprost, bimatoprost, travoprost, tafluprost, latanoprostene bunod), betablockers (timolol, metipranolol, carteolol), carbonic anhydrase inhibitors (dorzolamide, brinzolamide, acetazolamide, methazolamide), alfa-agonists (brimonidine, lopidine) and Rho kinase inhibitors (netarsudil). If the monotherapy does not reach the target pressure, a second drug with different action mechanism can be added [146,147].

However, novel treatment approaches using lipid nanoparticles are being studied, and they are summarized in Table 1.

The current pharmacological treatment with lipid nanoparticles is focused on the lowering of IOP. For this reason, some conventional drugs such as timolol, methazolamide and brimonidine have been encapsulated into SLNs or NLCs, obtaining better drug penetration with fewer topical administrations [147,148,149].

SLNs loading timolol showed a high and sustained permeation compared with timolol solution [147]. Similarly, methazolamide-loaded SLNs exhibit an enhancement of transcorneal permeation compared to commercial eye drops of methazolamide. Furthermore, SLNs of methazolamide are well tolerated in vivo because they are not irritating and do not show any histological changes in rabbit eyeballs [148]. Conventional first-line therapy, such as brimonidine, can also be encapsulated into SLNs and NLCs. In a comparative study between commercial eye drops, SLNs and NLCs loading brimonidine, it was revelated that NLCs presented higher therapeutic efficacy than SLNs and commercial eye drops because a prolonged IOP reduction was obtained [149].

Other conventional active compounds encapsulated into SLNs are betaxolol and pilocarpine. Betaxolol is a selective β_1_-receptor blocker and calcium channel blocker. In this sense, Hou and colleagues developed SLNs of betaxolol, which exhibited a controlled release and showed no damage to the cornea and conjunctiva after a log-term irritation test [150]. Furthermore, pilocarpine is also a conventional drug which is not commonly used due to the undesirable intense side effects such as miosis and myopia. To avoid pilocarpine side effects, SLNs were fabricated [151]. In this area, Lüfti and Müzeyyen prepared pilocarpine-loaded SLNs with the objective of maximizing the encapsulation of the drug and optimizing physicochemical parameters. Furthermore, the formulations showed a good stability for six months stored at 4 °C.

On the other hand, new compounds are being investigated as novel treatment for glaucoma with different action mechanisms than the ones currently on the market. Among them, melatonin is a neurohormone which is also synthesized in the eye. It is hypothesized that melatonin influences IOP rhythms [97]. For this reason, Leonardi et al. synthetized SLNs loading melatonin which were able to prolong the pre-corneal residence time, and their topical application decreased the IOP after a single administration with sustained action up to 24 h. Furthermore, the in vivo results showed a good ocular tolerability.

### 5.5. Retinitis Pigmentosa

Retinitis pigmentosa is a group of diseases which are hereditary and degenerative, characterized by pigmented deposits in the retina. An early clinical symptom is night blindness, often starting in childhood, followed by progressive loss of peripheral vision which can conclude in loss of central vision and complete blindness by midlife. These features evidence the degeneration of both photoreceptors, rods and cones. There is no pharmacological treatment for retinitis pigmentosa, but there are a few clinical trials with supplements of vitamin A and E, and they observed a slower progression of the disease [152,153,154,155].

In this area, S. Patel et al. prepared lipid nanoparticles containing an mRNA to treat posterior eye diseases, such as retinitis pigmentosa [156]. They applied subretinal injections to mice, and the results showed that the gene delivery was cell-type specific, with the majority of expression in the RPE and limited expression in the Müller glia. The gene delivery into the posterior segment of the eye resulted in an RPE expression sustained for 72 h and decreased by 120 h post-delivery, constituting a suitable vehicle for the treatment of retinitis pigmentosa.

**Table 1:** New treatment approaches for AMD, macular edema induced by AMD, diabetic retinopathy, uveitis and glaucoma using lipid nanoparticles.

Disease	Drug Encapsulated	Mechanism of Action	Lipid Nanoparticle	Administration Route	Preparation Method	Excipients	Physicochemical Characteristics	Reference
AMD	Atorvastatin	Protection of vascular intima of arteries. Prevents modification, oxidation and aggregation of some proteins of the eye	SLN	Eye drops	Hot high-pressure homogenization	Compritol^®^ 888 ATO PEG 400 Phospholipon 90H	Size: 256.3 ± 10.5 nm PDI: 0.26 ± 0.02 EE: 94.00 ± 1.21%	[132]
	Pentamethyl-6-chromanol	Antioxidant Protects RPE	SLN	-	High-shear homogenization	-	-	[133]
	Lutein	Prevent or limit the retinal damage by filtering out phototoxic short-wavelength visible light. Antioxidant	SLN	-	Hot homogenization and cold dilution method	Gelucire^®^ 44/14	Size: 79.70 nm	[92]
Macular edema induced by AMD	Dexametasone	Corticoid	Multilayer lipid membrane	Intravitreal injection	-	-	-	[134,135]
Diabetic retinopathy	siRNA silencing HuR expression	Inhibits the abnormal increase of VEGF in the retina	SLN	Intravitreal injection	Quasi-emulsion solvent diffusion	Softisan^®^ 100 Didecylmethylamonium bromide Tween^®^ 80	Size: 214 nm PDI: 0.177 ± 0.014 ZP: 44.9 ± 8.8 mV	[139]
	Etoposide	Anticancerous drug (retinoblastoma)	SLN	Intravitreal injection	Melt-emulsification and ultrasonication technique	Gelucire^®^ 44/14 Compritol^®^ 888 ATO Polysorbate 80 Mannitol	Size: 239.4 ± 2.3 nm PDI: 0.261 ± 0.001 EE: 80.96 ± 2.21%	[89]
	Epalrestat	Aldose reductase inhibitor	SLN	Contact lenses	Solvent diffusion method	Monostearin PEG_2000_-SA Pluronic^®^ F68	Size: 127–173 nm PDI: 0.115–0.195 ZP: 19–23 mV EE around 79%	[157]
Uveitis	Triamcinolone acetonide	Corticoid	NLC	Eye drops	Hot microemulsion method	Capmul^®^ MCM C10 Soya lecithin Captex^®^ 200 P Transcutol^®^ P Polysorbate 80 Stearylamine	Size: 198.9 ± 12.8 nm PDI: 0.326 ± 0.04 ZP: 35.8 ± 1.94 mV EE: 88.14 ± 3.03%	[94]
	Triamcinolone acetonide	Corticoid	SLN	*In situ* gel	Homogenization coupled with ultra-probe sonication method	Compritol^®^ 888 ATO Glyrecryl monostearate Pluronic^®^ F68 Polysorbate 80	Size: 200–350 nm PDI: 0.3–0.45 ZP: −52.31–(−64.35) mV EE: 97–99%,	[117]
Glaucoma	Timolol	Betablockers	SLN	Eye drops	Melt emulsification high pressure homogenization	Phospholipon 90G	Size: 37–47 nm Low PDI EE: 44%	[148]
	Methazolamide	Carbonic anhydrase inhibitors	SLN	Eye drops	Emulsion-solvent evaporation	Chitosan Lipoid S100 Polysorbate 80 PEG400	Size: 252.8 ± 4.0 nm PDI 0.1–0.2 ZP: 31.3 ± 1.37 mV EE: 58.3 ± 3.6%	[149]
	Brominidine	Alfa-agonists	SLN and NLC	Eye drops	High shear homogenisation method	Glyceryl monostearate Poloxamer^®^ P 188 Castor oil	NLC size 151 nm SLN size 198 nm NLC PDI 0.230 SLN PDI 0.496 NLC ZP −44.2 mV SLN ZP −39.2 MV NLC EE 83.6% SLN EE 82.5%	[150]
	Betaxolol hydrochloride	Selective β1-receptor blocker and calcium channel blocker	NLC	Eye drops	High shear homogenisation method	Phosphatidylcholine Glycerol monostearate Polysorbate 80 PEG 400 Sodium deoxycholate Mannitol Benzalkonium bromide	Size:150 nm PDI: 0.219 ZP: −18.86 mV EE: 75.2%	[158]
	Bimatoprost	Prostaglandin analogue	SLN	In situ gel	High shear homogenization	Glyceryl monostearate Polysorbate 80	Size 148.4–243.4 nm PDI 0.156–0.549 ZP of −19.3 mV EE 44.58–83.5%	[90]
	Pilocarpine	Muscarinic agonist	SLN	Eye drops	Quasi-emulsion solvent evaporation	Gelucire^®^ 44/14 Octadecylamine Polysorbate 80 Benzalkonium chloride	Size ˃ 500 nm PDI ˃ 0.6 ZP 46 mV EE ˃ 50%	[151]
	Melatonin	Decrease IOP by different mechanisms (IOP rhythm)	NLC	Eye drops	Quasi-emulsion solvent diffusion	Softisan^®^ 100 Polysorbate 80 Palmitic acid Stearic acid Didecyldimethylammonium bromide	Size 170–839 nm PDI around 0.2–0.3 ZP −3.0–61.0 mV EE of 83–96%	[51]
	Travoprost	Selective agonist for the FP prostanoid receptor	SLN and PEGylated SLN	Contact lenses	Solvent evaporation method	Compritol^®^ 888 ATO Lutrol^®^ F68 DSPE-PEG_2000_ Soy lecithin	Size 221–295 nm PDI < 0.3 ZP −20.4–(−38.8) mV EE ˃ 85%	[159]
	Latanoprost	Reduces the IOP by increasing the uveoscleral outflow	PEGylated SLN	Contact lenses	Solvent evaporation method	Precirol^®^ ATO 5 Pluronic^®^ F127 PEG_2000_-SA Soy lecithin	Size 104–150 nm PDI < 0.2 ZP −26–(−33) mV EE > 85%	[160]
Retinitis pigmentosa	mRNA	Achieve high gene expression while eliminating unintended genomic integration	PEGylated lipid nanoparticle	Subretinal injection	Microfluidic mixing	Dimethyldioctadecylammonium Cholesterol DMG-PEG_2000_ Mixture of lipids	Size < 200 nm PDI < 0.2 EE > 90%	[156]

## 6. Challenges in Ocular Drug Delivery to the Posterior Segment of the Eye

Lipid nanoparticles are an innovative drug delivery system which allows us to overcome the physiological barriers of the eye [80]. For this reason, lipid nanoparticles represent a novel treatment for posterior segment of the eye.

On the other hand, liposomes became the first nanosystem in US Food and Drug Administration (FDA) clinical trials. One example of clinical trials using liposomes as a drug delivery system was the evaluation of topical ophthalmic liposomes formulation carrying triamcinolone acetonide 0.1–0.2% (COFEPRIS 173300410A0035/2017) [161]. They synthesized liposomes containing triamcinolone acetonide as an active compound, Kolliphor HS 15^®^ (Polyethylene glycol [13]-hydroxystearate) and polyethylene glycol-12 glyceryl dimyristate. The result of the clinical trial was that liposomes were effective as a combined therapy for the prevention of macular edema associated with femtosecond laser-assisted cataract surgery. Another example of liposomes in clinical trials is the phase I/II trial of patients with macular edema due to retinal vein occlusion (NCT02006147) treated with dexamethasone sodium phosphate liposomes (ProDex^®^). The clinical trial resulted in signs of efficacy in both the reduction of retinal central subfield thickness and improvements in visual acuity [162].

The success of clinical trials using liposomes as a drug delivery system for ocular administration represents a challenge for lipid nanoparticles, which overcome the stability problems of liposomes [163]. There are not available clinical trials of lipid nanoparticles for ophthalmic administration, but there are few pre-clinical studies of lipid nanoparticles for drug delivery to the posterior segment of the eye, as specified in the previous sections. Furthermore, in clinical practice, the standard procedure in treating posterior segment of eye disorders is the intravitreal administration of drugs, leading to the disadvantages such as retinal detachment, iritis, or uveitis [23].

Despite the lack of clinical trials for lipid nanoparticles, one of the first patents for their use to the treatment of posterior eye diseases, such as diabetic retinopathy or macular degeneration, was developed in 2005 by Gasco M. (WO2005120469) [164]. SLNs prepared by dilution of a hot microemulsion were used as vehicles of nucleic acids or oligonucleotides. They can be applied topically on the eye surface and were able to release the drug in the posterior segment [6]. Moreover, another patent of chitosan-modified methazolamide SLN for the treatment of glaucoma (CN102793672A) has also been published [165]. In this case, nanoparticles were coated with chitosan to obtain a better stability and higher corneal permeability.

The development of effective ocular drug delivery system to treat posterior diseases faces many challenges due to the anatomy and physiology of the eye. Lipid nanoparticles could represent a powerful and effective tool for posterior eye diseases due to their unique properties. Moreover, after the success of liposomes in clinical trials, it opens a window to lipid nanoparticles.

## 7. Conclusions

The eye is formed by highly protective anatomical and physiological barriers which impose major obstacles against drug penetration to the ocular inner tissues. Therefore, the majority of conventional eyedrops do not allow the achieve of therapeutically active levels of drugs for the treatment of posterior eye diseases such as AMD, diabetic retinopathy, glaucoma and uveitis. However, there are other administration routes that allow us to reach inner tissues, but these routes present important drawbacks, including high side effects. In recent decades, lipid nanoparticles (SLNs and NLCs) have been designed to enhance bioavailability of the drugs into the inner tissues of the eye. Several advantages of these nanosystems include their physiological and biodegradable composition, good ocular tolerance and the possibility of upscale production. A high number of SLNs and NLCs have been developed, encapsulating several drugs, which opens a window for the treatment of posterior eye diseases employing safe and effective drug delivery systems. Furthermore, additional preclinical and clinical data regarding lipid nanoparticles for the posterior ocular segments will be necessary in order to obtain suitable treatments targeted towards the back part of the eye.

## Data Availability

Not applicable.
